# Efficacy of intravitreal dexamethasone implant used as monotherapy for the treatment of macular edema in non-infectious uveitis: a retrospective analysis

**DOI:** 10.1186/s12348-023-00360-3

**Published:** 2023-09-18

**Authors:** Rishi B. Gupta, Julius Ilin, Chloe C. Gottlieb

**Affiliations:** 1 Faculty of Medicine, University of Ottawa, Ottawa, ON Canada; 2 Ottawa Hospital Research Institute, Ottawa, ON Canada; 3 University of Ottawa Eye Institute, Ottawa, ON Canada

**Keywords:** Dexamethasone, Dexamethasone Intravitreal Implant, Macular Edema, Non-infectious Uveitis

## Abstract

**Background:**

Uveitic macular edema is a complication of acute or chronic uveitis. Current treatment regiments frequently have numerous side effects, often requiring supplemental treatment. This study investigates the efficacy of dexamethasone (DEX) intravitreal implants as monotherapy for treatment of macular edema in non-infectious intermediate, posterior or panuveitis.

**Methods and results:**

Retrospective chart review of thirty patients with intermediate, posterior and panuveitis treated with DEX. Outcomes measured were central retinal thickness (CRT) and best corrected visual acuity (BCVA). Baseline measurements of CRT and BCVA were measured within 1 month prior to intravitreal DEX implant and follow up measurements were collected until 1 year post implant. 48 implants on 39 eyes of 30 patients were included in this study; 64.1% of patients had an improvement in BCVA and 65.4% had a reduction in CRT. BCVA improved from 0.285 logMAR (SD: 0.312) at baseline to 0.175 logMAR (SD: 0.194) at 1 month and was sustained thereafter. Preliminary CRT data showed a decrease from 392 $\mu m$ (SD: $161\mu m$) at baseline to 303 $\mu m$ (SD: $80\mu m)$ at 1 month and 313 $\mu m$ (SD: $44\mu m)$ at 12 months.

**Conclusions:**

The DEX implant as monotherapy for macular edema in non-infectious uveitis was associated with a reduction in CRT and improvement in BCVA. The DEX implant, used as a monotherapy in eyes with intermediate, posterior and panuveitis, has the potential to treat uveitis without oral corticosteroid or other immunomodulatory therapy.

## Background

Uveitis accounts for 5%-20% of legal blindness in high income countries, particularly affecting the middle-aged population [1]. For decades, the first-line treatment of noninfectious uveitis has been corticosteroids administered orally, topically, or through either a periocular or intravitreal injection. Additional therapies that could be considered are oral corticosteroids, steroid-sparing immunosuppressive and biologic medications. Although effective in reducing inflammation within the vitreous humor, systemic corticosteroids are often associated with significant side effects [2]. These include elevated blood pressure, weight gain, glucose intolerance, osteopenia, avascular necrosis, bone marrow suppression and mood disturbances [3]. The blood-retina barrier, is composed of two layers, the outer layer is located at the level of the RPE cells and the inner layer is located at the level of endothelial cells from retinal vessels. Many forms of uveitis require high-dose corticosteroids over long periods of time for chronic uveitis, thereby further predisposing patients to further systemic side effects. Recent advances in nanotechnology saw the development of a sustained-release dexamethasone intravitreal implant which allows for direct corticosteroid delivery in the posterior segment of the eye; this study is designed to address the unmet medical need of adequate treatment of macular edema in patients with uveitis [4].

Few have investigated the efficacy and safety of the dexamethasone intravitreal implant as treatment of macular edema in noninfectious uveitis. Studies by Palla et al. and Lowder et al. demonstrated a significant improvement in visual acuity, as well as a significant reduction in central retinal thickness at 6 weeks post-implant when the implant was used in conjunction with stable systemic therapy [4, 5]. Similarly, Tsang et al. have demonstrated its effectiveness as an adjunct to conventional systemic corticosteroid treatment [6]. However, Brady et al. were unable to conclude that intravitreal implants were superior to traditional systemic steroidal therapy [1]. Furthermore, Thibaud et al. had concluded that the implant can treat macular edema effectively, but this study is limited in its clinical application due to adjunct systemic or topical therapy [7]. With such limited evidence, we hope to assess the efficacy of the dexamethasone intravitreal implant (DEX) as monotherapy for the treatment of macular edema in noninfectious uveitis. The DEX implant is a biodegradable copolymer of lactic acid and glycolic acid which is inserted into the vitreous and gradually releases 350 or 700 ug of dexamethasone within the eye for up to 6 months after insertion (Allergan Inc, data on file, 2006–2007). This study assessed the DEX implant as monotherapy to decrease CME for patients with intermediate, posterior and panuveitis, and provides novel and statistically significant evidence that the DEX implant can successfully be used without concomitant immune suppressive or oral corticosteroid.

## Materials and methods

### Patient selection

A retrospective chart review of consecutive patients with a single or multiple 0.7 mg DEX implant as first treatment for uveitis with macular edema was conducted. Patients who had received at least one DEX implant between June 1st, 2013 and July 31st, 2019 for macular edema associated with intermediate, posterior or panuveitis were selected. Patients included in this study had macular edema secondary to known uveitis irrespective of the level of activity of uveitis. The implant was administered by one of the authors (CG) at a tertiary institution using the manufacturer’s instructions. This study has been approved by the Ottawa Health Science Network Research Ethics Board and all research adhered to the tenets of the Declaration of Helsinki.

Any patients who had received concomitant oral prednisone or systemic immunomodulatory treatment (ie. systemic immunomodulatory therapy for uveitis would include methotrexate, cyclosporine, mycophenolate mofetil or mycophenolic acid and/or biologics such as Humira (adalimumab), Remicade (infliximab) or others) were excluded. Patients with infectious uveitis were excluded. Patients who had been administered periocular steroid or antiVEGF less than six weeks prior to the dexamethasone implant administration were also excluded. Furthermore, macular edema as a result of diabetes, retinal vein occlusion and other ocular conditions, as well as patients with intraocular surgery in the past 6 months or an IOP of > 21 mmHg at baseline were excluded.

### Data collection

The patients’ visit prior to insertion of the implant was used as baseline for data collection. Subsequent data collection was recorded from visits conducted at approximately 1, 4, 7, 10 and 12 months after each injection. At each visit, a spectral-domain optical coherence tomography (SD-OCT, Spectralis HRA + OCT, Heidelberg Engineering, Heidelberg, Germany) was performed after dilation, in addition to an ophthalmologic assessment, including BCVA, slit-lamp biomicroscopy, IOP, and binocular indirect ophthalmoscopy. Patients were followed longitudinally at the same clinic site with the same equipment. Macular edema was diagnosed by clinical examination and a CRT > 300 μm with the presence of typical cystic lesions on SD-OCT. Uveitis was graded according to Standardization of Uveitis Nomenclature (SUN) criteria and BCVA was converted to a logarithm of the minimum angle of resolution (log-MAR) units for statistical analysis [8]. Post-implantation complications, such as the development of cataract or increased IOP were also recorded. Success in BCVA was defined as an increase in BCVA of 0.1 logMAR units, or a 5-letter gain. Successful treatment with respect to CRT was defined as a return to 250 μm or a reduction of 100 μm from baseline.

### Statistical analysis

The main outcomes that were measured were changes in CRT on SD-OCT and BCVA measured with an ETDRS eye chart. The BCVA was converted to the logarithm of the minimum angle resolution (logMAR) for statistical analysis. Microsoft Excel was used to conduct statistical analysis. All two-tailed *p*-values were determined using a two-sample assuming unequal variances t-test. A Kaplan–Meier estimator was used to determine survival from relapse, which was defined as the need for re-implantation or an increase in retinal thickness by > 10%, and at least 50 μm since these levels are both clinically meaningful and above the measurement error between examinations. A *p* value < 0.05 was considered significant and 95% confidence intervals (CI) were calculated using standard methods.

## Results

### Patient demographic data

Forty-eight implants were placed in 39 eyes of 30 patients with non-infectious intermediate, posterior or panuveitis who met the inclusion criteria for this study. 20 females and 10 males were selected with a mean age of 47.3 (range 19–77). 35 of 39 eyes meet the criteria for active non-anterior posterior uveitis according to the SUN criteria prior to treatment with the DEX implant [8]. Nine eyes required a second implant within 12 months and one eye developed a cataract 7 months post-operatively.

The etiology of uveitis in the patients included the following: idiopathic uveitis (*n* = 22), sarcoidosis (*n* = 5), autoimmune retinopathy (*n* = 1) and birdshot retinopathy (*n* = 1). Data summarized in Table 1 confirms that none of the patients were receiving any concomitant systemic immunomodulatory therapy during the treatment with the DEX implant.

**Table 1:** Patient uveitis baseline description and details

Etiology	Number of patients
Idiopathic	22
Sarcoidosis	5
Autoimmune	1
Birdshot Retinopathy	1
Uveitis Site	Number of eyes
Intermediate	31
Posterior	7
Panuveitis	1
Vitreous Cells/Haze	Average (min—max)
Vitreous Cells	1.3 (0—2)
Vitreous Haze	0.74 (0—3)

### Best corrected visual acuity

**Fig. 1:**
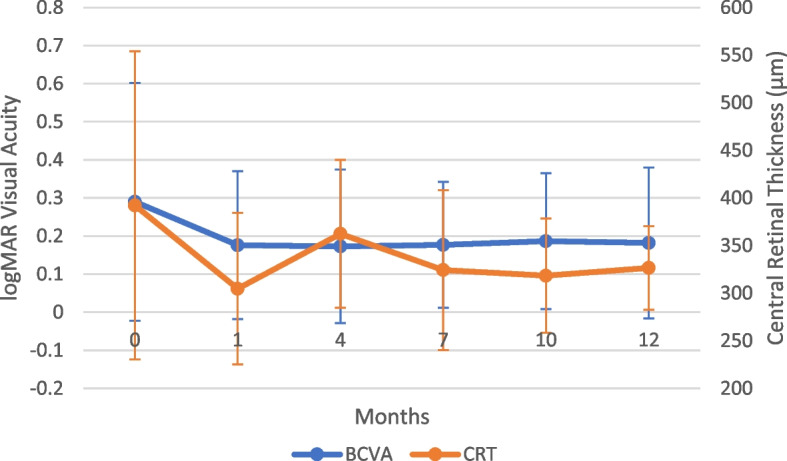
Effect of dexamethasone implant on best-corrected visual acuity. Average BCVA (logMAR) and central retinal thickness (CRT, µm) of study eyes during the course of follow-up. Error bars represent one standard deviation from the mean

All BCVA data is included in Table 2 and patients with multiple BCVA data points are graphically depicted in Fig. 1. At baseline, the patients had an average BCVA value of 0.285 logMAR (SD: 0.312). One month after implant, this level improved to 0.175 logMAR (SD: 0.194, *p* = 0.06) (Fig. 1). This improvement was then sustained throughout the course of the rest of the treatment with no statistically significant change in value (*p* = 0.87). The *p*-values listed above are changes relative to baseline at 0 months (Table 2). The ETDRS equivalent improvement was approximately from 20/40 to 20/32. 5 eyes had a successful improvement in BCVA but not in CRT due to persistent intraretinal fluid.

**Table 2:** Descriptive analysis of central retinal thickness and best corrected visual acuity post-implant insertion

	Baseline (mean (SE) N)	One month post implant (mean (SE) N)	Four months post implant (mean (SE) N)	Seven months post implant (mean (SE) N)	Ten months post implant (mean (SE) N)	Twelve months post implant (mean (SE) N)
CRT ($\mu m$)	392 (32) *n* = 26	303 (22) *n* = 13	362 (25) *n* = 10	324 (24) *n* = 12	318 (16) *n* = 14	313 (14) *n* = 10
BCVA (logMAR)	0.285 (0.05) *n* = 39	0.175 (0.03) *n* = 37	0.169 (0.04) *n* = 34	0.176 (0.03) *n* = 30	0.172 (0.04) *n* = 17	0.186 (0.05) *n* = 14

### Central retinal thickness

All CRT data is included in Table 2 and patients with multiple CRT data points are graphically depicted in Fig. 1. 65.4% (17 or 26 collected eyes) had a reduction in CRT. The mean CRT at baseline was 392 μm (SD: 162 μm) which decreased to 303 μm (SD: 80 μm, *p* = 0.03). At 4 months, the CRT increased to 362 μm (SD: 78 μm, *p* = 0.46). At 7 and 10 months, the values dropped to 324 μm (SD: 84 μm, *p* = 0.10) and 318 μm (SD: 60 μm, *p* = 0.05), respectively. The improvement was then sustained thereafter (Fig. 1). The *p*-values listed above are changes relative to baseline at 0 months (Table 2). One eye had successful treatment with respect to CRT but not in BCVA.

### Intraocular pressure

**Fig. 2:**
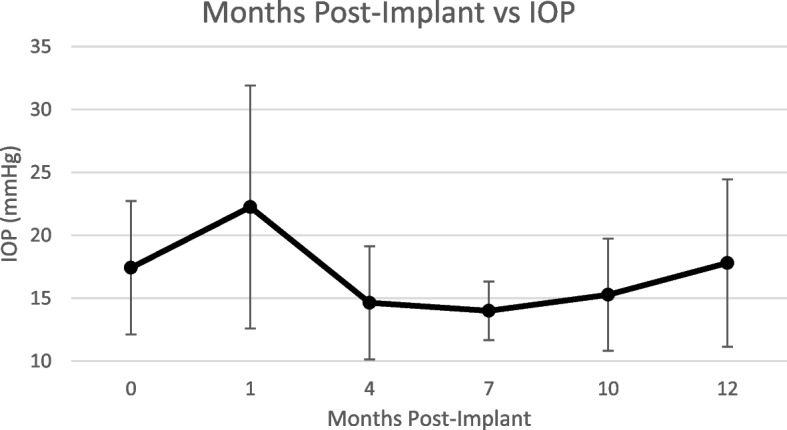
Post-implant intraocular pressure changes over 12 months. The baseline IOP was 17.42 mmHg (SD: 5.30) and increased to 22.25 mmHg (SD: 9.64) at 1 month. Subsequently, it decreased to 14.64 mmHg (SD: 4.49) at 4 months. The IOP returned to baseline of 15.28 mmHg (SD: 4.46) at 10 months and was sustained thereafter

Intraocular pressure was measured at each month post-operatively. Although the average IOP increased from 17.42 mmHg (SD: 5.30) at baseline to 22.25 mmHg (SD: 9.64) (*p* = 0.01) at 1 month, it dropped significantly at 4 months to 14.63 mmHg (SD: 4.49) (*p* = 0.02). It returned to baseline at 10 months with a value of 15.27 mmHg (SD: 4.46) (*p* = 0.12) and was sustained thereafter (Fig. 2). The *p*-values listed above are changes with respect to baseline at 0 months (Table 3).

**Table 3:** Change in intraocular pressure during the months following dexamethasone implantation

Month Post-Implant	0	1	4	7	10	12
IOP (mmHg (SE) N)	17.42 (0.86) *n* = 38	22.25 (1.6) *n* = 36	14.64 (0.78) *n* = 33	14 (0.44) *n* = 28	15.28 (1.05) *n* = 18	17.8 (2.1) *n* = 10

### Duration of effect and cataract formation

**Fig. 3:**
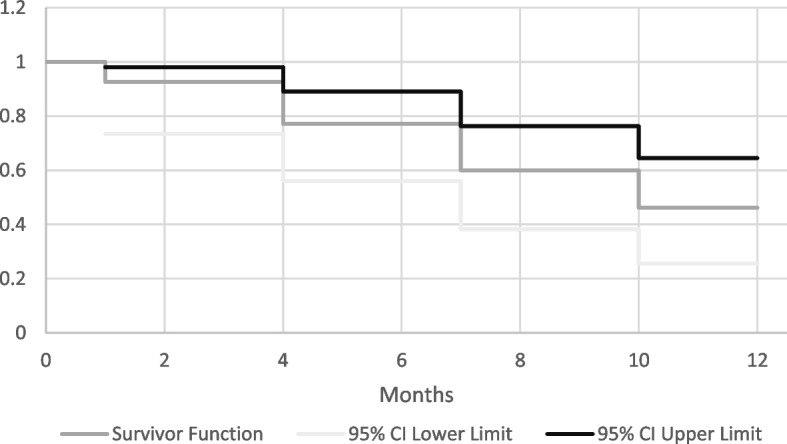
Duration of dexamethasone implant effect. Kaplan–Meier survival plot showing the duration of effect of the initial dexamethasone implant. The average time to relapse after one injection was 7 months. Estimates of treatment success were 77% between 4 and 7 months and 46% after 10 months. Failure was defined as a follow-up with an increase in CRT of > 10% and at least 50 μm from baseline or the need for a repeat implant within 12 months post-operatively

In order to determine the duration of the effect of dexamethasone implants, time to failure was recorded. Failure was defined as a follow-up with an increase in CRT of > 10% and at least 50 μm from baseline or the need for a repeat implant within 12 months post-operatively. The need for a repeat implant was assessed based on recurrence of uveitis. One patient developed a cataract at 7 months, and this was considered as a potential complication. The Kaplan–Meier estimate of treatment success was 77% between 4 to 7 months and 46% was sustained beyond 10 months (Fig. 3).

## Discussion

In 2011, the DEX implant was approved for the treatment of posterior non-infectious uveitis based on a prospective randomized clinical trial by the HURON Study group [4]. In their study, 47% of the eyes achieved the primary outcome measure of a vitreous haze score of 0 at 8 weeks [4]. The DEX implant, however, has remained off-label for the treatment of macular edema in non-infectious uveitis. Macular edema is one of the leading causes of blindness and accounts for up to one-third of blindness and visual impairment in chronic non-infectious uveitis [9]. Two studies have been conducted regarding the use of multiple DEX implants in macular edema caused by non-infectious uveitis [10, 11]. In both of these studies, there was a statistically significant decrease in CRT on OCT at 1 month and up to 6 months from the first injection, with a median time to re-injection of 6 months [10, 11]. Only one of the studies compared the effect of multiple implants to the first implant, and no significant difference in effect was observed [11].

We demonstrated that the DEX implant, when used in conjunction with systemic corticosteroids, improved BCVA and reduced CRT in patients with CME secondary to non-infectious uveitis in our previous 2017 publication [6]. A literature review was conducted to search for previous studies assessing the use of the DEX implant as a monotherapy and we were unable to find any such studies. Monotherapy would avoid the use of concomitant systemic immune suppression and also avoid side-effects from systemic corticosteroid treatment, while maintaining adequate vision. In our study, the etiology of uveitis in the majority of the patients was attributed to idiopathic uveitis. The etiologies may result in varying responses to the implant and this may be a confounding variable in the results analysis. This study shows that the DEX implant, used as a monotherapy without concomitant or recent prior systemic treatment to effectively treat macular edema in non-infectious uveitis, with these results showing a statistically significant improvement in both BCVA and CRT over the course of 12 months. BCVA improved on average from 0.285 logMAR units to 0.175 logMAR units. From a practical perspective, the difference in logMAR is an improvement in patient vision from 20/40 to 20/32.

CRT decreased from 392 μm to 318 μm during the same time period. Between 1 month post-operatively and 12 months, there was no statistically significant difference in CRT reduction, showing that improvement in CRT occurred within one month and was unchanged thereafter. It was noted at month four that CRT returned close to baseline levels as the implant is expected to last 4–6 months. The rise in CRT at 4 months may reflect the nine eyes (9/39, 23%) who had recurrence of macular edema and then required a second implant.

A Kaplan–Meier survival analysis was conducted to study the survival of the treatment during the 12-month analysis period. A 77% success between 4 to 7 months and 46% beyond 10 months was demonstrated, providing strong evidence for the DEX implant as treatment for macular edema in uveitis. There were 5 eyes that had a successful improvement in BCVA and only one eye that had a successful improvement in CRT but not in BCVA.

Furthermore, only one of the eyes developed a cataract and none of the patients required systemic corticosteroid treatment. IOP may be elevated in patients with ocular implants and this is often treated by topical therapy [6], however, our study demonstrated that despite initial increases in IOP at one month post-implant, the pressure dropped significantly 4 months post-implant. These pressure values even returned to normal after 10 months and were sustained thereafter (Table 3). Patients did not receive any systemic corticosteroid or immunosuppressive therapy; nine of the 39 (23%) eyes required a second implant over a study period of 12 months. The data suggests that either patients did not have aggressive uveitis, or the dexamethasone implant was effective. This provides preliminary evidence to support the hypothesis that DEX implants can be used as monotherapy for macular edema in uveitis, however, keeping in mind that the patient’s underlying inflammatory disease would need to be addressed with other systemic treatments.

The current standard of care for posterior non-infectious uveitis involves systemic corticosteroids or the use of any of the following local treatments: intravitreal and periocular steroid injections, topical steroids and intravitreal anti-VEGF agents [12]. In situations when patients have adverse reactions or treatment-resistant uveitis, managing this condition presents as a significant challenge. This retrospective study displayed a significant reduction in CRT and improvement in BCVA, similar to previous studies which have been published regarding the use of the 0.7 mg dexamethasone implant for this condition [12–14].

The study's limitations include data collection from a single centre, referral bias to a tertiary care centre, and small cohort size. A confounding variable is the prior use of topical corticosteroids, which was not controlled or standardized among patients. The idiopathic etiology of uveitis was disproportionately high with most patients having idiopathic uveitis. The group of patients enrolled in this study had inflammatory posterior segment disease; one had retinal vasculitis and none had occlusive vasculopathy. Treatment outcomes could be impacted, and future studies may include controlling for the etiology of the uveitis. Long term studies of the effects of single versus multiple implants and a direct comparison to systemic corticosteroids would reveal if the DEX implant is equivalent to systemic corticosteroid in patients with posterior segment non-infectious uveitis. Lastly, data points at each time point decreased progressively; the results presented in this study, however, can be interpreted to assess trends, showing a significant decrease in CRT and increase in BCVA, thereby demonstrating that the implant can lead to both anatomical and visual improvements for patients.

This study has provided initial evidence with statistical significance that the DEX implant can successfully be used as monotherapy to treat uveitic macular edema.

## Conclusion

The DEX implant as monotherapy for macular edema in non-infectious uveitis was associated with a significant reduction in CRT and a statistically significant improvement in BCVA, with only 10.2% of patients requiring additional treatment in a 12-month period. In this study, patients had not received any prior systemic corticosteroids or immunosuppressive therapy. Our study shows that the DEX intravitreal implant, used as a monotherapy in eyes with intermediate, posterior and panuveitis is an effective treatment for macular edema.

## Data Availability

The datasets used and/or analyzed during the current study are available from the corresponding author on reasonable request.

## References

[1] Brady CJ, Villanti AC, Law HA (2016). Corticosteroid implants for chronic non-infectious uveitis. Cochrane Database Syst Rev.

[2] Yap YC, Papathomas T, Kamal A (2015) Results of intravitreal dexamethasone implant 0.7 mg (Ozurdex®) in non-infectious posterior uveitis. Int J Ophthalmol. 10.3980/j.issn.2222-3959.2015.04.34. Published online. 10.3980/j.issn.2222-3959.2015.04.34 PMC4539658 26309888

[3] Hunter RS, Lobo AM (2011). Dexamethasone intravitreal implantfor the treatment of noninfectious uveitis. Clin Ophthalmol.

[4] Lowder C, Belfort R, Lightman S (2011). Dexamethasone intravitreal implant for noninfectious intermediate or posterior uveitis. Arch Ophthalmol.

[5] Palla S, Biswas J, Nagesha C (2015). Efficacy of Ozurdex implant in treatment of noninfectious intermediate uveitis. Indian J Ophthalmol.

[6] Tsang AC, Virgili G, Abtahi M, Gottlieb CC (2017). Intravitreal Dexamethasone Implant for the Treatment of Macular Edema in Chronic Non-infectious Uveitis. Ocul Immunol Inflamm.

[7] Mathis T, Cerquaglia A, Weber M, et al (2021) Uveitis treated with dexamethasone implant. Retina. 41(3). 10.1097/IAE.0000000000002901 10.1097/IAE.0000000000002901 32618834

[8] Jabs DA, Nussenblatt RB, Rosenbaum JT (2005). Standardization of uveitis nomenclature for reporting clinical data. Results of the first international workshop. Am J Ophthalmol..

[9] Okhravi N, Lightman S (2003). Cystoid macular edema in uveitis. Ocul Immunol Inflamm.

[10] Zarranz-Ventura J, Carreño E, Johnston RL (2014). Multicenter study of intravitreal dexamethasone implant in noninfectious uveitis: Indications, outcomes, and reinjection frequency. Am J Ophthalmol.

[11] Tomkins-Netzer O, Taylor SRJ, Bar A (2014). Treatment with repeat dexamethasone implants results in long-term disease control in eyes with noninfectious uveitis. Ophthalmology.

[12] Cao JH, Mulvahill M, Zhang L, Joondeph BC, Dacey MS (2014). Dexamethasone intravitreal implant in the treatment of persistent uveitic macular edema in the absence of active inflammation. Ophthalmology.

[13] Lightman S, Belfort R, Naik RK (2013). Vision-related functioning outcomes of dexamethasone intravitreal implant in noninfectious intermediate or posterior uveitis. Investig Ophthalmol Vis Sci.

[14] Miserocchi E, Modorati G, Pastore MR, Bandello F (2012). Dexamethasone intravitreal implant: An effective adjunctive treatment for recalcitrant noninfectious uveitis. Ophthalmologica.

